# Hepatitis A and E Viruses in Mussels from Cherrat Estuary in Morocco: Detection by Real-Time Reverse Transcription PCR Analysis

**DOI:** 10.1155/2022/8066356

**Published:** 2022-11-28

**Authors:** Hanaâ Bazir, Najwa Hassou, Fatiha El Mellouli, Hasnae Zekhnini, Saliha Najib, Moulay Mustapha Ennaji

**Affiliations:** ^1^Team of Virology Oncology and Biotechnology, Laboratory of Virology Oncology Biosciences Environment and New Energies, Faculty of Sciences & Technologies Mohammedia, University Hassan II of Casablanca, Casablanca, Morocco; ^2^Casablanca Regional Research and Analysis Laboratory of National Office of Food Safety (ONSSA), Casablanca, Morocco; ^3^Geology-Biology-Geography (GBG), Polydisciplinary Faculty of Khouribga, Sultan Moulay Slimane University, Beni Mellal, Morocco

## Abstract

The aim of the present study was to evaluate hepatitis A virus (HAV) and hepatitis E virus (HEV) contamination in mussels (*Mytilus galloprovincialis*) from Cherrat estuary (Moroccan Atlantic Coast), Morocco. In total, 52 samples (*n* = 12 mussels/each) were collected at four sites in the estuary, monthly, between March 2019 and March 2020. HAV and HEV were detected by real-time reverse transcription polymerase chain reaction (RT-PCR) according to the ISO/TS 15216 method. HAV was detected in 46.15% of analyzed samples. Conversely, HEV was not detected in any sample. Moreover, the HAV detection rate was significantly associated with seasonal rainfall variations. This qualitative study on HAV and HEV contamination highlights the interest of studying mussel samples from wild areas. As HAV presence in mussels represents a potential health risk, viral contamination surveillance of mussels is necessary to protect consumers. HAV shellfish contamination must be monitored at Cherrat estuary because of the role played by shellfish as HAV reservoirs and/or vehicles in fecal-oral HAV transmission.

## 1. Introduction

Shellfish is a major component of the global aquatic food [1]. This term describes crustaceans and mollusks. Mollusks are subdivided into cephalopods, gastropods, and bivalves. Commercially important bivalve mollusks are mussels, oysters, clams, and scallops [2]. Mussels are organisms that feed by filtering water from the aquatic environment [3]. Therefore, they can concentrate microbial contaminants [3–5], including enteric viruses [6–8]. Moreover, mussels can live on sand/gravel sediments where enteric viruses persist in large quantities in the surface layer in contact with water [9, 10] and can remain infectious for several months [11, 12]. In the majority of cases of infection, different viral strains have been detected in the implicated shellfish, suggesting contamination in their original environment by wastewater [13–17]. Indeed, entropic activities near the coast have a major impact on shellfish quality [18]. Pollution of coastal waters can lead to contamination of bivalve mollusks [4], and their consumption (raw or undercooked) is associated with human viral diseases [19, 20]. Importantly, enteric viruses are often identified during enteric disease outbreaks caused by the consumption of shellfish that meets the bacteriological standards [16, 21–24].

Bivalve mollusks can be the main vector for transmission of enteric diseases [25], caused by hepatitis A virus (HAV) [26], norovirus [27], hepatitis E virus (HEV) [28], adenovirus, rotavirus, enterovirus, and astrovirus [29–32]. These viruses can cause gastroenteritis and viral hepatitis [33]. Viral hepatitis of enteric origin is caused by HAV and HEV, two viruses encountered epidemically, and even endemically, in countries with poor sanitation [34].

HAV is 28–30 nm in diameter, nonenveloped, 7.5 kb single-stranded, linear, positive-sense RNA virus, classified in the genus *Hepatovirus* within the *Picornaviridae* family [35]. This virus is classified into six genotypes (I–VI), of which genotypes I–III can infect humans [36]. HAV causes a self-limiting disease, without serious complications in most cases, and does not lead to chronic infection or chronic liver infection. However, fulminant hepatitis and death have been reported [37]. HAV is present worldwide, and the seroprevalence rate depends on hygiene conditions [38]. In developing countries (i.e., Asia, Africa, Latin, and Central America), HAV infection is highly endemic, and infected people are often asymptomatic [36].

HEV is a non-enveloped, 7.2 kb single-stranded, positive-sense RNA virus belonging to the genus *Orthohepevirus* and the *Hepeviridae* family [39–41]. Each year, ∼20 million people are infected by HEV worldwide [7, 42], mainly in developing countries [43]. HEV causes self-limiting acute hepatitis; however, extrahepatic manifestations (neurological and renal damage) and chronic infection, especially in immunocompromised patients and organ transplant recipients, are increasing, thus representing an important clinical problem [42]. Moreover, pregnant women can develop fulminant hepatic failure, with a mortality rate of up to 30% [44]. Although HEV has only one serotype, it shows great genetic diversity with four major genotypes (from 1 to 4) in the *Orthohepevirus A* species [39, 40, 45]. Genotypes 1 and 2 have been exclusively isolated in humans and are predominant in Asia, Africa, and Central America [41, 46, 47]. They have been implicated in major human epidemics due to consumption of HEV-contaminated water or food in countries with poor sanitation [39].

Morocco has put in place a national strategy for the protection and sustainable development of the environment [48]. In the framework of this strategy, Oued Cherrat is categorized as one of the priority sites of biological and ecological interest due to its ecological, social, and economic importance and also the strong pressure by human exploitation that is causing important alterations. Within this strategy, a protection program has been put in place to accompany this site development through the rational use of resources (including shellfish) and ecotourism [49]. In this context, this article presents the first environmental and virological study carried out at Cherrat estuary (Casa-Settat region in Morocco) to monitor viral contamination of mussels and the associated risk factors for a year.

The study objectives were as follows:

- To determine the contamination rate by HAV and HEV in mussels harvested at different sites at Cherrat estuary
- To study the correlation between seasonal factors and the mussel contamination rate by HAV and HEV

## 2. Materials and Methods

### 2.1. Study Area

Cherrat estuary (33°49′52.71″°N—7°07′23.33″°W) is located on the Moroccan Atlantic coast in the province of Benslimane (Casa-Settat region), 53 km north of Casablanca. It is a transition area between the Cherrat beach and Oued Cherrat, a site of biological and ecological interest [49]. The beach is classified as category A for bathing water [50]: less than 100/100 mL amount of fecal coliforms (*Escherichia coli*) and enterococci (fecal streptococci), according to the relevant national standard (NM 03.7.200), transposed from the European Directive (76/160/EEC) and the Directives WHO/UNEP, applicable to the health surveillance of marine bathing waters.

Water quality in this area is affected by watershed runoff, urban wastewater management malfunctions, and overflows of nearby sewerage systems.

**Figure 1:**
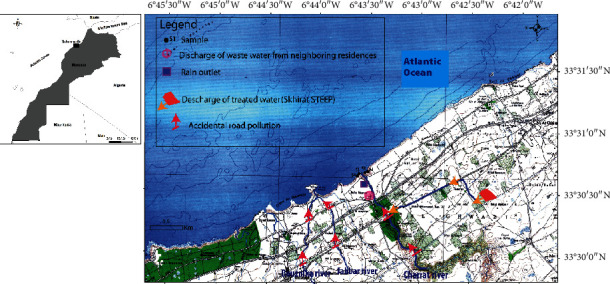
Mussel sampling areas at Cherrat estuary.

Mussels were collected at four sites (*S*1, *S*2, *S*3, and *S*4) at Cherrat estuary (Figure 1):

- *S*1 and *S*2, located on the right rocky side of Cherrat estuary (with a distance step of 100 m)
- *S*3 and *S*4, located on the rocky left side of Cherrat estuary (with a distance step of 100 m)

These sites correspond to an overexploited wild mussel harvesting area. These mussels are sold locally in an informal and traditional way by the local population, without any sanitary control.

### 2.2. Mussel Collection

Naturally growing mussels (*Mytilus galloprovincialis*) were collected monthly at the four sites at Cherrat estuary over a 13-month period (from March 2019 to March 2020). In total, 52 samples (*n* = 12 mussels/sample) were collected. All samples were shipped to the laboratory in a refrigerated box within 24 h after collection.

### 2.3. Sample Processing and RNA Extraction

Mussel samples were processed following the ISO/TS 15216-1:2017 (Microbiology of the food chain-horizontal method for determination of HAV and norovirus using real-time RT-PCR-part 1: method for quantification) protocol for HAV detection in food samples.

Mussels were rapidly rinsed, shucked, and dissected. The hepatopancreas was removed and pooled to have a final weight of (2 ± 0.2) g for each sample. Digestive tissues were mixed with 10 *μ*L of mengovirus (CeeramTOOLS®) as a nucleic acid extraction control. This was followed by incubation with proteinase *K* solution (2.0 ± 0.2 mL) at (37 ± 1.0)°C with shaking at 320 rpm for (60 ± 5) min to digest the tissues and a second incubation at (60 ± 2.0)°C for (15 ± 1) min in a waterbath. Then, samples were centrifuged at 3000*g* at room temperature for (5.0 ± 0.5) min, and the supernatants were collected in clean tubes.

Viral RNA was extracted from 500 *μ*L of each supernatant using the Nucleospin RNA virus kit (Macherey Nagel Germany) according to the manufacturer's instructions and eluted in 100 *μ*L of elution buffer.

### 2.4. RT-PCR Assays

#### 2.4.1. HAV Detection

The Luna® Universal Probe One-Step RT-qPCR Kit (New England BioLabs) was used for HAV and mengovirus MC_O_ strain detection by real-time RT-PCR. Briefly, 4 *μ*L of each RNA sample was amplified in 21 *μ*L of reaction mix that contained 1X reaction mix, 10 *μ*L of the Luna Universal Probe One-Step Reaction mix (2X), 0.5 pmol/*μ*L of forward primer, 0.9 pmol/*μ*L of reverse primer, 0.25 pmol/*μ*L of probe, and 1.25 *μ*L of Luna Warm Start® RT Enzyme Mix (20X). Primers and probes and the respective references are listed in Table 1.

The amplification protocol was as follows: reverse transcription reaction at 55°C for 10 min, initial denaturation at 95°C for 1 min, followed by 45 cycles of denaturation at 95°C for 10 s, and extension at 60°C for 1 min.

Viral RNA extracted from each sample was amplified in duplicate using 4 *μ*L undiluted and ten-fold diluted sample to evaluate the presence of inhibitors, using the mengovirus analysis according to the manufacturer's instructions, by comparing the Ct values of the undiluted and diluted RNA samples. A Ct value difference <3.3 indicated the presence of inhibitors.

For each run, a mengovirus standard curve was generated using a ten-fold serial dilution. Extraction efficiency was evaluated by comparing the Ct value of the mengovirus RNA extracted from the samples to this standard curve. Results ≥1% were considered valid. A sample was considered positive when the Ct value was ≤33 in at least two replicates, without evidence of amplification in the negative controls.

RNA extracted from the HAV HM 175 strain was used as a positive control. Two negative controls were included in each RT-PCR series: negative control of the extraction with the PCR mixture and negative control of the amplification (nuclease-free water with the PCR mixture).

#### 2.4.2. HEV Detection

The genesig real-time PCR detection kit from Primerdesign™ Ltd. supplied by oasig lyophilized One-Step RT-qPCR MasterMix was used for HEV detection (based on the detection of the ORF2 capsid protein-encoding gene). 5 *μ*L of each RNA sample was amplified in 20 *μ*L of reaction mix that contained 1X reaction mix, 10 *μ*L of oasig™ lyophilized One-Step 2X RT-qPCR Master Mix, 1 *μ*L of HEV primer/probe mix, 1 *μ*L of internal extraction control primer/probe mix, and 3 *μ*L of RNase/DNase-free water. The primers have been described by the manufacturer in the genesig® Advanced Kit handbook. The amplification protocol was as follows: reverse transcription at 55°C for 10 min, enzyme activation at 95°C for 2 min, followed by 50 cycles of amplification with denaturation at 95°C for 10 s.

Each sample was amplified in duplicate (undiluted and 1/10 diluted). Two negative controls were included in each RT-PCR run: negative control of the extraction with the PCR mixture and negative control of the amplification (RNase/DNase-free water with the PCR mixture). The positive control should have a Cq value between 16 and 23.

Five standards (from 2 × 10^4^ copy numbers/*μ*L to 2 copy numbers/*μ*L) were prepared by dilution of the positive control (2 × 10^5^ copy numbers/*μ*L) for quantitative analysis. The absence of PCR inhibitors was tested using the internal RNA extraction control from the RT-qPCR kit. Briefly, 4 *μ*L of the internal extraction control RNA was added to each RNA sample, and the Ct values were compared with the Ct value of the negative control containing the internal control. The internal control was detected through the VIC channel and gave a Cq value of 28 ± 3.

All RT-PCR tests to detect HAV and HEV were performed on an AriaMx (Agilent Technologies, Santa Clara, CA) 96-well plate real-time PCR instrument. Fluorogenic data were collected at 60°C for 60 s through the FAM and VIC channels, using the Agilent Aria software v 1.71.

### 2.5. Statistical Analysis

Spearman's rank correlation analysis was used to correlate the results of positive samples pooled by month and pluviometry. These correlations were performed with the total accumulated rain of the previous month (obtained from the Bouregreg and Chaouia hydraulic basin agency) assuming that each mussel harvesting area was mainly affected by the rain of the preceding month. All statistical analyses were performed using the statistical package SPSS Statistics 17.0. Statistical significance was determined by a *P* value <0.05.

## 3. Results

For this study, all samples provided valid results with acceptable extraction efficiencies (>10%). HEV was not detected in any of the collected wild mussel samples. Among the 52 samples analyzed, 24 were HAV-positive (46.15%). HAV contamination rate was lowest at site *S*1 (3/13; 23.07%), compared with sites *S*2, *S*3, and *S*4 (46.15%, 61.54%, and 53.85%, respectively) (Table 2).

Fisher's exact test showed significant differences in contamination rates between the dry period (May to September) and the rainy period (October to April) (*P* < 0.0001). A significant positive correlation between the number of positive samples and the average rainfall was observed.

## 4. Discussion

Shellfish are known to accumulate human pathogens, such as human enteric viruses (e.g., rotavirus in mussels [52, 53], enterovirus [32, 54], HAV [26, 55], and norovirus [27, 56, 57] in oysters).

In this study, only HAV, but not HEV, was found in the collected mussel samples. The difference in contamination rates among the four sites may be explained by their different location and the influence of currents or other hydrographic variables. However, more studies are needed to test these hypotheses. Our results are in agreement with previous studies [58–60] reporting HAV presence in mussels collected at local markets, a culture farm, and during environmental monitoring.

HAV presence in mussels at Cherrat estuary is explained by the strong persistence of the virus in the environment [61] or by a continuous supply of viruses from runoff from the watershed of the Oued Cherrat, urban wastewater, malfunctions, and overflows from the sewage system that is not up to standard [50]. This can induce diffuse pollution and affect the estuarine environment where mussels naturally develop [62].

Regarding HEV, some studies on shellfish did not detect HEV in mussels marketed in France [63], Italy [64], Denmark, [65], and Thailand [66]. On the other hand, several studies reported HEV presence in mussels grown naturally in wild areas and commercially available in Scotland [28], Spain [7, 67, 68], and Italy [4, 69]. Similarly, there is evidence that HEV is circulating in the Moroccan population [70]. The absence of detection at Cherrat estuary is explained by the number of HEV particles discharged in the environment is too low to be detected or the virus may have a very short period of persistence in human waste. More work on HEV persistence in human waste is needed to better evaluate this point.

## 5. Conclusion

Our study showed that HAV contamination rate in mussels from Cherrat estuary was quite high and significantly associated with seasonal variations. Therefore, updating the existing legislation to include viral hazards in the monitoring of bivalve mollusk harvesting areas is an urgent matter for the competent authorities. In addition, illegal harvesting of mussels from this area must be better controlled due to the threat to public health caused by the risk of viral contamination. Monitoring the presence of enteric viruses in bivalve mollusks may contribute to the prevention of viral food poisoning and the promotion of public health.

## Figures and Tables

**Table 1:** Sequences of the primers and probe used for HAV detection.

Virus	Sequence	Reference
HAV	Forward primer HAV68	[51]
	5′-TCA CCG CCG TTT GCC TAG-3′	
	Reverse primer HAV240	[51]
	5′-GGA GAG CCC TGG AAG AAA G-3′	
	Probe HAV150	[51]
	FAM 5′-CCT GAA CCT GCA GGA ATT AA-3′ MGBNFQ	

**Table 2:** HAV and HEV contamination rate in mussels collected at four sites at Cherrat estuary.

Month	Rainfall (mm)	HAV				HEV			
		*S*1	*S*2	*S*3	*S*4	*S*1	*S*2	*S*3	*S*4
March 2019	26.8	−	+ (Ct 29.69)	−	+ (Ct 32.78)	−	−	−	−
April 2019	34.5	−	−	+ (Ct 31.75)	−	−	−	−	−
May 2019	45.6	−	−	-	+ (Ct 33.07)	−	−	−	−
June 2019	10.9	−	−	+ (Ct 30.43)	−	−	−	−	−
July 2019	0	−	−	+ (Ct 32.53)	−	−	−	−	−
August 2019	0	−	−	−	−	−	−	−	−
September 2019	5	−	−	−	−	−	−	−	−
October 2019	10.1	+ (Ct 33.42)	+ (Ct 33.26)	+ (Ct 33.23)	+ (Ct 33.23)	−	−	−	−
November 2019	32.6	−	+ (Ct 32.75)	+ (Ct 32.42)	+ (Ct 33.10)	−	−	−	−
December 2019	74.8	+ (Ct 32.83)	+ (Ct 33.09)	+ (Ct 32.59)	+ (Ct 30.38)	−	−	−	−
January 2020	270	+ (Ct 33.23)	+ (Ct 30.72)	+ (Ct 31.75)	+ (Ct 33.41)	−	−	−	−
February 2020	58.4	−	−	+ (Ct 31.16)	+ (Ct 32.13)	−	−	−	−
March 2020	18.5	−	+ (Ct 32.78)	−	−	−	−	−	−
		**3/13 (23.07%)**	**6/13 (46.15%)**	**8/13 (61.54%)**	**7/13 (53.85%)**	**0 (0%)**	**0 (0%)**	**0 (0%)**	**0 (0%)**
		**24/52 (46.15%)**				**0 (0%)**			

## Data Availability

The data used to support this study are included within the article.
